# Oral lymphomatoid granulomatosis, the first sign of a ‘rare disease’: a case report

**DOI:** 10.1186/1752-1947-8-152

**Published:** 2014-05-15

**Authors:** Pasqualino Cargini, Maria Civica, Laura Sollima, Emanuela Di Cola, Emanuele Pontecorvi, Tommaso Cutilli

**Affiliations:** 1 Department of Life, Health & Environmental Sciences - Maxillofacial Surgery Operative Unit and Postgraduate School of Maxillofacial Surgery, University of L'Aquila, Via della Comunità Europea, 13, 67100 L’Aquila, Italy; 2 Department of Life, Health & Environmental Sciences, Postgraduate School of Pathology, University of L’Aquila, L'Aquila, Italy

**Keywords:** Lymphomatoid granulomatosis, Oral involvement, Rare diseases

## Abstract

**Introduction:**

Lymphomatoid granulomatosis is an uncommon Epstein-Barr virus-positive B-cell lymphoma, an angiocentric-destructive process with a predominant T-cell background. Lymphomatoid granulomatosis is listed among rare diseases. Common localization is in the lungs. Lymphomatoid granulomatosis with oral involvement is described in only two reports. In this report, we describe a third case of oral lymphomatoid granulomatosis.

**Case presentation:**

A 65-year-old Caucasian man with a gingival ulceration underwent a biopsy. The histological pattern was compatible with a grade III lymphomatoid granulomatosis. The staging revealed a nodular lesion in the lower lobe of his right lung. Our patient also presented with hemoptysis, an unusual and not reported clinical sign. Rituximab, cyclophosphamide, doxorubicin, vincristine, and prednisone chemotherapy was performed every three weeks for six cycles.

**Conclusions:**

The pulmonary nodule and the gingival lesion disappeared. At eight-month follow-up, our patient is disease-free. We wish to emphasize that the oral manifestation described was the first sign of the disease and allowed for diagnosis. This case report adds to the medical literature for the particular clinical presentation of this rare disease.

## Introduction

Over 90 percent of humans are infected by Epstein-Barr virus (EBV) and the infection persists for life. EBV infection is associated with a number of malignancies and can infect B cells, T cells and natural killer (NK) cells. The head and neck region contains several compartments (that is nasopharynx, nasal and paranasal sinuses and so on) each of which is potentially affected by neoplastic lymphoid proliferation [1]. Lymphomatoid granulomatosis (LYG) is an angiocentric-destructive process characterized by EBV-infected B cells and T-cell reaction. Common localizations are lungs and mediastinum nodes. Less frequently, LYG has also been described in other sites. Oral localization is very infrequent. In the scientific literature, we found that LYG with oral involvement in immune-competent individuals has only been described in two reports: a palatal involvement, a local recurrence of primary pulmonary LYG [2]; and a gingival lesion, the only site of the disease, considered an atypical form [3]. We describe the third case of LYG with oral involvement and we wish to emphasize that the oral manifestation described was the first sign of the disease and allowed for the diagnosis of the simultaneous pulmonary LYG.

## Case presentation

**Figure 1:**
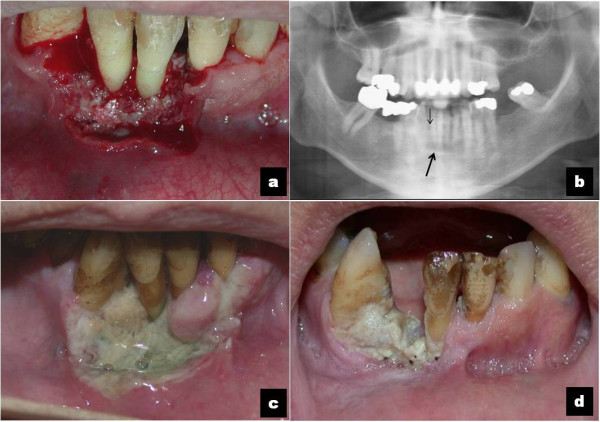
**The course of the clinical presentation of the oral lymphomatoid granulomatosis before and after therapy. (a)** Reddened, easy to bleed gingival ulceration with granulomatous tissue, dental root exposure and mobility of teeth 4.1 and 4.2. **(b)** Radiological evaluation: panoramic radiography shows mandibular alterations in the area of the incisors (black arrow) and resorption of the right incisor-canine alveolare ridge (thin black arrow). Axial computed tomography scans shows osteitis (black arrow). **(c)** The gingival ulceration has extended, with bacterial colonization. **(d)** At eight-month follow-up: the granulomatous lesion has disappeared; the gingival inflammation persists with the spontaneous loss of tooth 4.2.

**Figure 2:**
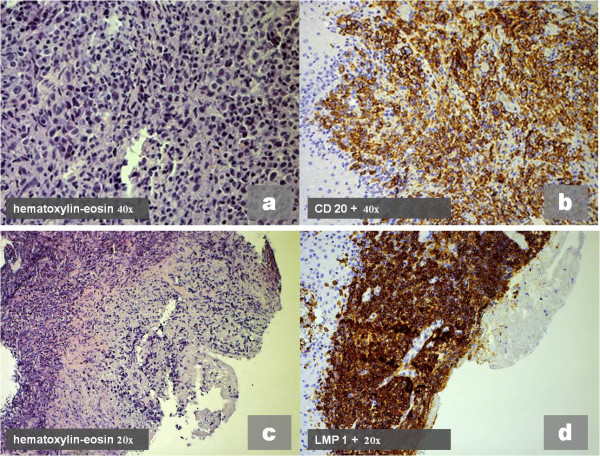
**Histologic pattern of lymphomatoid granulomatosis: (a) (c) Rich B-cell blasts (hematoxylin and eosin 40×).** Immunohistochemical study: **(b)** wide expression of CD 20+ receptor (20×); **(d)** high positivity for latent membrane protein 1, expression of Epstein-Barr virus infection in B cells and their activation (20×).

**Figure 3:**
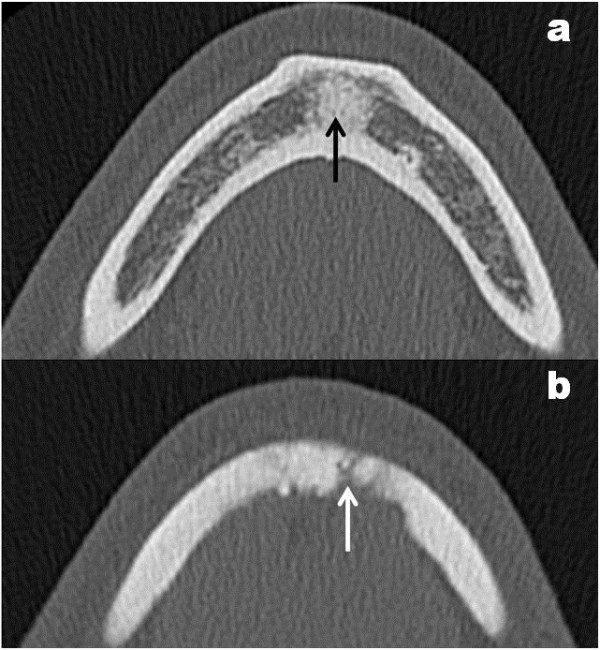
**Computed tomography axial scans of the mandible.** Computed tomography axial scans of the mandible show **(a)** the aspect of the thickening osteitis of the anterior arch (black arrow) and **(b)** the irregular bone resorption (white arrow).

**Figure 4:**
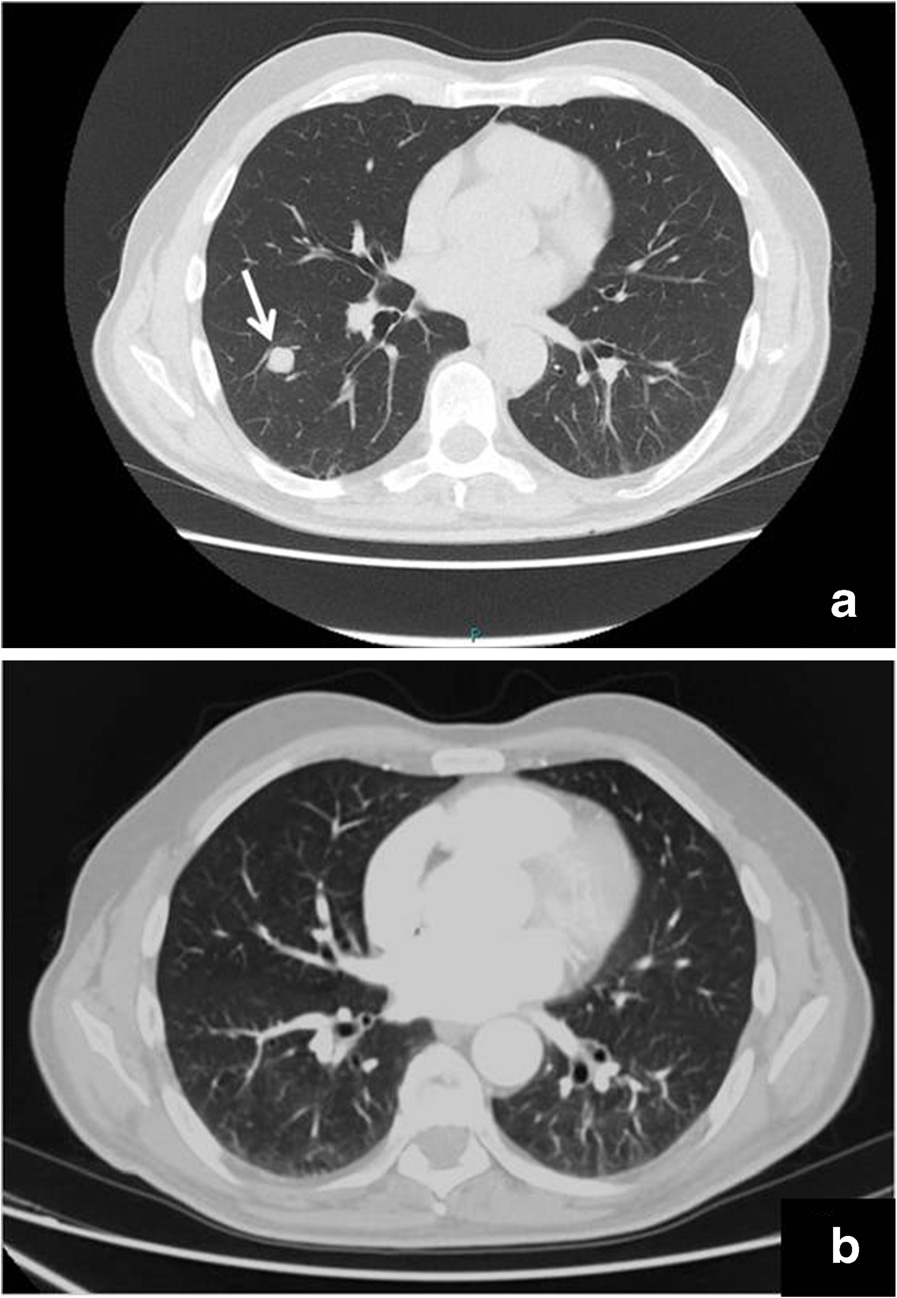
**Total-body computed tomography scans with contrast enhancement.** Total-body computed tomography scans show: **(a)** a nodular mass (a ‘cannonball’ - 13x14 mm) in the lower lobe of the right lung (white arrow); **(b)** at eight-month follow-up, the scan shows the disappearance of the pulmonary lesion.

A Caucasian 65-year-old man, suffering for a month from a persistent pain in the inferior oral fornix and in the symphysis region of the mandible, was hospitalized. Our patient did not have a relevant medical history: no use of tobacco, no alcohol abuse, no medications, no weight loss. During an oral examination, very poor oral hygiene was observed with severe widespread periodontal disease, dental plaque, and several exposed dental roots, especially in the inferior dental arch. In this context, a severe gingival ulceration extending from the 3.1 to the 4.3 region was observed with mobility of teeth 4.1 and 4.2. The lesion showed granulomatous tissue, and reddened swollen margins (Figure 1a). Our patient reported severe pain on palpation. No laterocervical nodes were present. Limited local bone resorption of the alveolar process was observed on standard radiologic examination (panoramic radiography) (Figure 1b). His routine laboratory test results were negative. A biopsy was performed immediately and our patient was discharged from the department with antibiotics (clavulanic acid 125mg and amoxicillin 875mg twice a day) and a prescription for nonsteroidal anti-inflammatory drugs. The histological report described a rich inflammatory infiltrate of CD3-, CD4- and CD8-positive small T cells, CD30-positive B cells, and a low number of CD56- and CD57-positive natural killer (NK) cells. The lymphoproliferative foci, CD20+, appeared to have angiocentric necrotizing blasts and high positivity for latent membrane protein 1 (LMP1) and EBV protein gene 3, compatible with grade III LYG (Figure 2). Our patient was immediately recalled and a total-body computed tomography (TBCT) scan with intravenous contrast enhancement was performed. The study of the mandible showed osteitis of the anterior arch characterized by thickening of the spongiosa (Figure 3a); it also showed irregular bone resorption at the site of the gingival lesion (Figure 3b). The TBCT scan detected a nodular lesion (13×14 mm) in the lower lobe of his right lung, described as a ‘cannonball’, which showed contrast enhancement due to profuse vascularization. No mediastinic nodes were present but multiple nodes were present in his right axillary fossae and peri-iliac veins bilaterally (Figure 4). No pathological enhancement was detected in his central nervous system (CNS). Our patient was evaluated at the Medical Oncology Service and began chemotherapy (ChT) treatment. In the meantime, the gingival ulceration had grown, with bacterial colonization (Figure 1b). Our patient also reported several episodes of hemoptysis. According to guidelines [2-6], rituximab, cyclophosphamide, doxorubicin, vincristine, and prednisone (R-CHOP), the ChT scheme, was administrated every three weeks for six cycles. A restaging with a TBCT scan was performed, respectively, after four cycles, six cycles and after eight months. The pulmonary nodule and the gingival lesion disappeared (Figures 4b,1d). The patient at the time of this writing was disease-free.

## Discussion

Lymphomatoid granulomatosis is an EBV-positive large B-cell lymphoma angiocentric-destructive process/disorder/disease with a predominant T-cell background, which is characterized by prominent pulmonary involvement; extra pulmonary sites described are kidneys, liver, skin and the CNS. LYG was first described by Leibow in 1972 [7] with diagnosis based on the following histological triad: 1) nodular polymorphic lymphoid infiltrate by small B cells, plasma cells and large atypical mononuclear cells; 2) angiitis due a full-thickness infiltration of vessels by lymphocytes; 3) granulomatosis with central necrosis within the lymphoid nodules.

LYG is listed among rare diseases [8]. Relevant data regarding prevalence and race predilection are unavailable. Elements that are known are the male/female ratio (2:1) and the mean age at presentation (the fifth to sixth decade of life). LYG is progressive and fatal. The mortality rate is estimated between 50 and 60 percent. A mean survival of 14 months to two years from diagnosis has been described in the literature [9]. Common signs of lung localization are cough, dyspnea and chest pain, observed in 54 to 80 percent of cases. Systemic involvement with fever, sweating and weight loss is described in 30 to 70 percent of cases. Cutaneous manifestations are observed in 36 to 53 percent of cases, consisting of erythema, nodules and plaques. In CNS involvement, there are deficit signs such as blindness, hemiparesis and ataxia in 10 to 35 percent of cases. Ulceration of the upper airways is described in 10 percent of cases. Therefore, LYG with oral involvement is a very rare event and occurs in apparently immunocompetent young subjects without modification of clinical and laboratory data. There is evidence in LYG of defective immunosurveillance with a low count of CD4/CD8 T cells. Due to the angiocentric nature and extranodal localization, LYG is confused with other angiocentric diseases such as NK/T-cell lymphoma, but EBV-infected B-cells in LYG is the main distinguishing factor of these two pathologic entities [2]. The immunohistological feature is described in the case presentation. The number of immunoblasts EBV infected is related to the grade and prognosis of LYG. Grade I lesions have a low number of atypical EBV + (<5 in high-power field) and necrosis areas are minimal. In grade II, the number of atypical cells infected is moderate (from 5 to 20 in high-power field) and necrosis areas are evident. In grade III, numerous atypical cells are identified (>50 in high-power field) and necrosis areas are prominent. Lower-grade (I to II) LYG occasionally show a spontaneous remission and the strategies applied to enhance the host's underlying immune system are effective. According to guidelines of the World Health Organization (WHO) classification, grade III LYG should be considered to be diffuse large B-cell lymphoma (DLBCL) (either as a not otherwise specified T cell/histiocyte-rich large B-cell lymphoma, or EBV-positive DLBCL of older people, eventually associated with the intake of immunosuppressive drugs) [10]. In some cases, it has an uncertain malignant potential; in other cases, it has a poor prognosis.

The National Cancer Institute has recommended a combination of immunotherapy and chemotherapy for grade III LYG treatment. R-CHOP, given every three weeks for six or eight cycles, is the chemotherapy scheme followed. After four cycles, restaging with a TBCT scan is necessary [11].

## Conclusions

LYG is a rare EBV-driven progressive lymphoproliferative disease. Epidemiological data are fragmented and incomplete, thus diagnosis is very difficult and requires correlation between clinical signs and histopathologic pattern. Our aim in presenting this case, still in observation, is that of indicating the possibility that LYG can begin in the oral cavity, and also of focusing attention on two other particular aspects: First, the oral involvement of LYG is a very rare event, but the gingival ulceration and its easy access has allowed for immediate biopsy, the early diagnosis of LYG and the early discovery of its pulmonary localization. It may be possible that oral and lung lesions are concomitant manifestations of LYG. Second, the unusual sign of hemoptysis - an alarm bell for pulmonary disease - is common in all lesions near bronchial ramifications and vessels, or indicates cavitation. Only one study described hemoptysis associated with LYG but this work reports multiple large cavitation areas of lung near the pulmonary artery [12].

Instead, in our report, a CT scan with contrast enhancement showed only one ‘cannonball’ nodule in the middle field of the right inferior lobe, distant from the bronchial lumen and major vessels, in absence of cavitation. Hemoptysis, therefore, should not have been present in the case we describe. However, one of the diagnostic criteria of LYG is a full-thickness angiitis by lymphocytes and it is possible to hypothesize that the angiitis is the basis of hemoptysis. In conclusion, we wish to emphasize that the oral manifestation described was the first sign of the disease and allowed for diagnosis and for the simultaneous pulmonary localization.

## Consent

Written informed consent was obtained from the patient for publication of this case report and any accompanying images. A copy of the written consent is available for review by the Editor-in-Chief of this journal.

## Abbreviations

ChT: chemotherapy; CNS: central nervous system; DLBCL: diffuse large B-cell lymphoma; EBV: Epstein-Barr virus; LMP1: latent membrane protein 1; LYG: lymphomatoid granulomatosis; NK: natural killer; R-CHOP: rituximab, cyclophosphamide, doxorubicin, vincristine, and prednisone; TBCT: total-body computer tomography; WHO: World Health Organization.

## Competing interests

The authors declare that they have no competing interests.

## Authors’ contributions

PC was a major contributor in writing the manuscript together with TC. MC and EP analyzed and interpreted the patient data regarding the rare disease. LS and EDC performed the histological examination. TC was a major contributor in writing the manuscript, together with TC, and followed and checked all the steps of the manuscript. All authors read and approved the final manuscript.
